# A Multimodal Adaptive Inter-Region Attention-Guided Network for Brain Tumor Classification

**DOI:** 10.1109/access.2025.3627777

**Published:** 2025-10-31

**Authors:** IBRAHIM ABDELHALIEM, JOSE DIXON, ABEER ABDELHAMID, GEHAD A. SALEH, FAHMI KHALIFA

**Affiliations:** 1 Department of Computer Science, Faculty of Computers and Information, Assiut University, Asyut 71515, Egypt; 2 Department of Bioengineering, University of Louisville, Louisville, KY 40292, USA; 3 Electrical and Computer Engineering Department, School of Engineering, Morgan State University, Baltimore, MD 21251, USA; 4 Electronics and Communications Engineering Department, Mansoura University, Mansoura 35516, Egypt; 5 Department of Diagnostic and Interventional Radiology, Mansoura University, Mansoura 35516, Egypt

**Keywords:** Brain tumor, multimodal, information retention, adaptive region attention, DW-MRI, T2-MRI

## Abstract

Accurate brain tumor classification is critical for ensuring timely and effective medical interventions. In recent years, artificial intelligence (AI)-driven diagnostic systems have emerged as transformative tools that optimize the classification process and enable rapid, objective decision-making. However, existing methods often suffer from limitations such as the loss of high-frequency details during multimodal preprocessing, inadequate cross-modal feature alignment, and insufficient focus on shared tumor regions within 3D architectures. To address these challenges, this study introduces a novel AI-based framework for advanced brain tumor classification. Specifically, we propose a multimodal magnetic resonance imaging (MRI) architecture that integrates Diffusion-Weighted MRI (DW-MRI) and T2-weighted MRI (T2-MRI) modalities, uniquely combining them in a dual-branch 3D neural architecture with advanced preprocessing and attention mechanisms. The preprocessing pipeline employs a learnable High-Frequency Information Retention (HFIR) technique to resize T2-MRI images, maintaining consistent spatial dimensions across modalities while preserving essential image details. The architecture utilizes dual-branch 3D convolutional neural networks (CNN) for modality-specific feature extraction, enhanced by a novel Adaptive Region Attention (ARA) module that dynamically aligns and emphasizes highly informative regions shared across modalities, providing deeper and more consistent insights into tumor characteristics. Rigorous evaluation on a dataset of brain MRI scans including three tumor classes demonstrates that the proposed framework achieves overall accuracy, sensitivity, and specificity of 92.86%, 80.00%, and 94.12%, respectively. Statistical analyses using bootstrap-resampled F1-scores confirm significant outperformance over other state-of-the-art models, underscoring its robust and interpretable potential for precise brain tumor diagnosis.

## I. INTRODUCTION

Cancer is the leading cause of death worldwide and hinders life expectancy. Brain tumors, a major type of cancer, result from abnormal cell growth in the brain, damaging vital tissues, influencing the brain or spinal cord, and advancing to cancer [1], [2]. The National Brain Tumor Society (NBTS) estimates that over 94K individuals will receive a primary brain tumor diagnosis in 2025 [3], [4] while approximately 1M Americans are currently living with a brain tumor [3]. Radiologists classify less aggressive and non-progressive tumors as benign (grades I and II). Originating in the brain, they develop slowly and cannot reach other parts of the body. Conversely, malignant tumors can appear in various forms and degrees. Meningiomas and gliomas (which combine astrocytomas, oligodendrogliomas, and ependymomas) are the most common brain tumors in adults. With 15 subtypes, the majority of which are benign and regarded as WHO grade I [5], meningioma is a single category in the WHO’s central nervous system (CNS)5thedition. Pituitaryadenomas are usually noncancerous, gradually developing masses, and represent the most prevalent form of pituitary gland tumors [6]. While pituitary tumors form around the pituitary gland, meningiomas grow around the skull area. Thus, early-stage brain tumor detection has become a crucial but difficult task for guiding therapy decisions, thus ensuring the appropriate option to save the patient’s life.

Prompt identification of brain tumors is critical for successful treatment. This information informs treatment plans and significantly impacts patient survival rates [7], [8]. However, due to their complex characteristics, medical practitioners find it difficult to diagnose brain tumors. Typically, doctors diagnose brain tumors using a set of physical and neurological tests. Biopsy, examined using various histological approaches, is the most consistent method for detecting brain cancer. However, this procedure is intrusive, time-consuming, subjective, inconsistent, and increases the risk of bleeding, tissue damage, and functional loss [9]. Given the rapid progression of cancer, where patient survival relies on precise and early diagnosis [10], [11], this is extremely concerning.

AI-based non-invasive tools effectively utilize various imaging techniques, such as magnetic resonance imaging (MRI) and computed tomography (CT), for diagnosis, followed by biopsy and pathological examination for confirmation. Among non-ionizing and non-invasive techniques, MRI is the most effective, in addition to its ability to provide detailed images of the brain’s anatomy [12]. Accurate interpretation of these images is a demanding task for identifying and classifying brain cancer. Radiologists’ experience has historically been the basis for this study; however, manual analysis is time-consuming, subjective, and prone to human error, especially in complex cases or among less experienced staff [13]. Modern neuroimaging has many new non-invasive tools made possible by artificial intelligence (AI). These tools help doctors understand the structure and function of brain tumors [9], [10], [14]. Recently, AI-based tools that streamline medical imaging have become crucial in determining the type and stage of tumors, as well as in developing treatment plans. Typically, T1-weighted (T1-w) contrast-enhanced MRI helps physicians distinguish primary tumors, such as meningiomas, and secondary tumors.

The objective of this study was to enhance brain tumor diagnosis using advanced AI techniques. We propose a multimodal MRI-based integrative tool with the potential to enhance precise diagnosis, leading to significantly more informed treatment. The proposed model integrates a combination of High-Frequency Information Retention (HFIR), 3D convolutional neural network (3D CNN) feature extractors, and Adaptive Region Attention (ARA) modules that have an unrivaled ability to comprehend both the nuanced details and the larger spatial contexts contained within the brain MRI images. Given the size differences between T2- and DWI-weighted MRIs, we incorporated an information retention strategy to prevent information loss during image downscaling. This strategy, in turn, preserves MRI details for better diagnosis. First, the HFIR module preprocesses T2-MRI images to preserve high-frequency information. Second, the model employs a 3D ResNet architecture for feature extraction that delves deeper into spatial relationships to capture the information required for accurate brain tumor analysis. Finally, the model integrates CNN-derived multimodal features using a novel attention module, the ARA, to accentuate common crucial features before classification. The latter obtains its representation through a Dropout layer, a 3D convolutional layer used as a classifier, a Rectified Linear Unit (ReLU) function, and 3D Adaptive Average Pooling (AAP). The proposed framework contributes significantly to the field of brain tumor classification in the following ways:

- We developed an integrative multimodal system that combines diverse learning modules to capture salient features while incorporating cross-domain knowledge, thereby providing valuable insights for brain tumor classification.
- The HFIR module is a learnable block that preserves intricate T2-MRI details while resizing for improved diagnosis.
- The framework designs a novel ARA module to emphasize key features shared between MRI modalities. This module, unlike global attention mechanisms in existing literature, complements the multi-branch 3D CNN model, enhancing its capacity to comprehend the interactions and relationships between different modalities thoroughly.

The paper organizes the remaining sections into four consecutive parts: related work, methodology, results and discussion, and conclusion. In the related work section II, we examine the current literature and methodologies developed for brain tumor classification, identifying gaps and opportunities for innovation. The methodology in section III describes the proposed architecture and its parameter settings. Section IV presents the quantitative and qualitative findings and discussion from experiments, which we validated using various ablation schemes and evaluation criteria. Finally, the conclusions in section VI summarize key findings, discuss implications, and propose avenues for future research.

## II. RELATED WORK

Medical image analysis, particularly brain disease detection, has seen significant advancements due to AI- and deep learning (DL)-based algorithms. Numerous studies have explored multimodal MRI inputs, CNNs, hybrid models, and attention-based mechanisms to improve accuracy and robustness. For instance, Sekhar et al. [15] combined traditional ML with a fine-tuned GoogleNet on the (GBCE)-MRI dataset, achieving more than 98% precision in glioma detection. Similarly, transformer-based models, such as Transformer-Enhanced CNN [16] and hybrid CNN attention models [17] have achieved high performance on datasets such as BRATS and Figshare. ResNet variants [18], correlation-based methods [19], and detection strategies using YOLO and GoogleNet [20] further demonstrate the versatility of DL architectures. Attention mechanisms, such as the MANet proposed by Shaik and Cherukuri [21], also enhance classification by focusing on critical features. Comparative analyses using popular architectures (e.g., VGG-16, ResNet-50, and Inception-v3) [22] and decision support systems using pre-trained DenseNet and SVM classifiers [23] highlight ongoing efforts to optimize accuracy and interpretability. EfficientNet-based transfer learning models [24] and multi-scale feature designs such as MultiFeNet [25] continue to push performance boundaries, with metrics above 98% in most cases. These studies provide a strong foundation for our study, which builds on the strengths of existing methods while addressing key limitations in feature representation and generalizability. Xu et al. [26] proposed a cross-modality guided ResNet backbone with dual attention for tumor grading, achieving strong performance across BraTS 2018 and 2019. However, the model exhibits modality-dependent variability, suggesting limited adaptability to diverse input combinations. Similarly, Guo et al. [27] and Fang and Wang [28] used dual-path or MMDNet-based segmentation approaches but faced constraints from small sample sizes or limited contextual awareness. Current multimodal models often lack consistent performance across modalities and underutilize spatial and structural complementarity between inputs such as T2 and DWI, especially in 3D.

Several studies, including those by Eitel et al. [29] and Juneja et al. [30] explored denoising and frequency preservation using CNN filters or autoencoder pipelines. Sarah et al. [31] and Sahu [32] employ handcrafted preprocessing techniques (e.g., SGLDM, LPIF) to boost performance. However, these methods do not explicitly aim to retain high-frequency diagnostic features during resolution reduction. Most focus on either denoising or traditional filters, not on channel-aware, learnable retention modules such as Pixel Unshuffle or frequency-aware fusion. Attention-enhanced networks, such as MANet [21], Tabatabaei et al. [17], and Hekmat et al. [33] improve saliency detection and feature weighting using channel or spatial attention layers. However, these typically operate at the global level and do not address the local tumor region focus or cross-modality region alignment. Region-specific attention, particularly across multimodal inputs, remains poorly developed. These models do not dynamically align spatial tumor cues across modalities, as the ARA module in our framework does.

Recent studies by Cao et al. [34], Wu et al. [35], and Li et al. [36] adopted 3D CNNs or dual-branch designs to improve volumetric context understanding. However, these models still face constraints, such as early fusion bottlenecks, 2D spatial reliance, and incomplete integration of 3D modality-specific branches. Our approach introduces parallel 3D ResNet branches for each modality, retaining modality-specific features and performing late fusion after the region-level alignment, enabling deeper semantic integration.

Prior studies have achieved promising results using CNNs, attention, and transformer-based models; however, most studies only use single-modality MRI or rely on standard fusion strategies with limited cross-modal integration. In contrast, our study introduces a multimodal framework that combines DW-MRI and T2-MRI using an HFIR technique and a novel ARA module. This design enables a more effective feature alignment across modalities and improves the focus on the tumor regions, which enhances diagnostic performance and helps clinicians interpret results more effectively.

While prior studies demonstrate strong performance, researchers often constrain their methods by using single-modality MRI inputs, relying on pre-segmented tumor regions, or basic early fusion strategies that fail to support effective cross-modal interaction. Even recent dual-branch and attention-based designs often overlook explicit high-frequency feature preservation or region-aware refinement.

To the best of our knowledge, no prior study has integrated the specific contributions of this study. In contrast, our proposed framework integrates DW-MRI and T2-MRI within a dual-branch architecture that includes an HFIR module and an ARA mechanism. This combination promotes enhanced cross-modal feature alignment, improved tumor localization, and greater robustness to modality-specific variations–ultimately contributing to both superior diagnostic accuracy and model interpretability.

Although they have drawbacks, most of the cited studies applied ML and DL techniques, which are effective in identifying brain cancer. The quality and quantity of data used in these algorithms determine their outputs. These algorithms may fail to detect complex tumor classes, including outstanding and uncommon brain tumor types. Similarly, some researchers deployed different ViT versions for brain tumor classification. Contrary to current research, solo CNN and ViT-based approaches for brain tumor classification show poor performance and require further development. Table 1 presents an overview of the examined literature focusing on brain tumor detection using MRI images. The Table summarizes the technical innovations, performance, and limitations of each related study.

## III. METHODOLOGY

**FIGURE 1.**
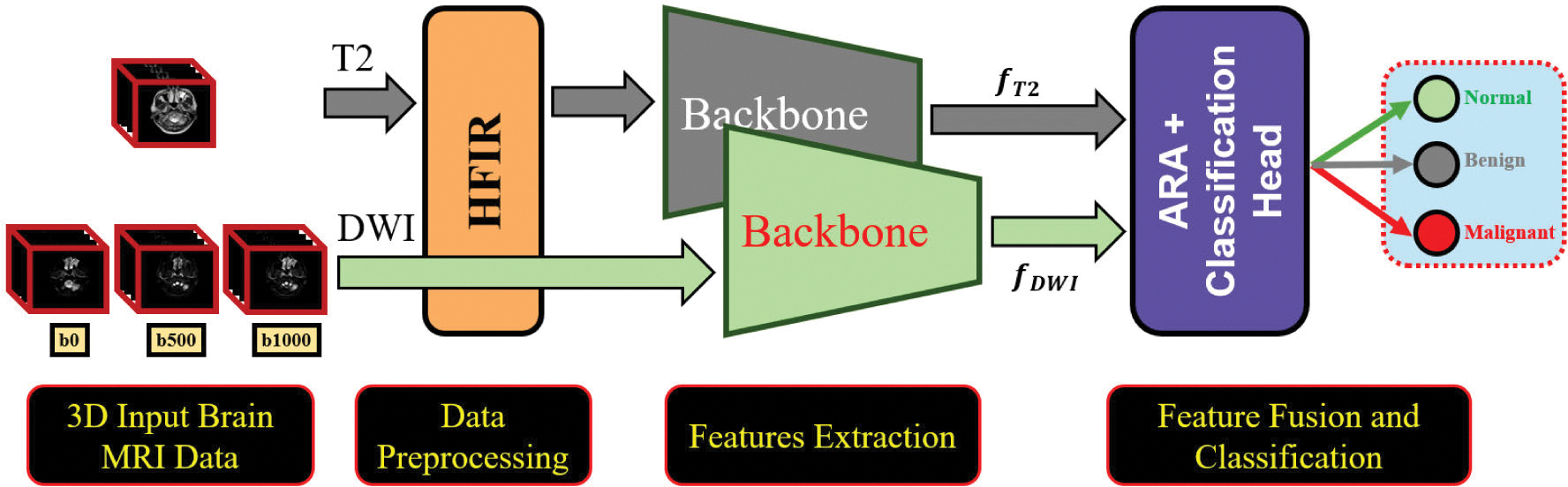
A general review of the suggested brain tumor classification architecture.

The proposed framework is a multi-step approach for brain tumor classification using MRI data. First, the HFIR module preprocesses the higher-resolution T2-MRI sequences to match the dimensions of the DW-MRI inputs, preserving essential high-frequency features during downsampling. Second, two separate backbone networks independently extract features from the $f_{T2}$ (HFIR-processed T2-MRI) and $f_{DWI}$ (raw DW-MRI) data. The framework then combines these extracted features via the ARA module. Finally, the classification head categorizes the brain MRI data as normal, benign, or malignant. Fig. 1 illustrates the workflow of the proposed framework for brain tumor diagnosis.

The dataset comprised multimodal MRI images, specifically DW-MRI and T2-MRI, with resolutions varying from 256 × 256 × 18 for DW-MRI to 512 × 512 × 18 for T2-MRI. The initial step in our pipeline involved resizing the T2-MRI images to match the dimensions of the DW-MRI images. Our model resizes the input data using the HFIR module, which preserves the essential high-frequency features needed for efficient learning. The HFIR module produced feature maps and forwarded them to the next stage, which involved a two-branch model. To improve feature extraction functionality, this model uses two 3D ResNet-18 networks: one to handle the HFIR module’s output (processed T2-MRI) and another to handle the DW-MRI images. The proposed ARA module helps distinguish between important and unimportant regions. The classification head receives the final result from the ARA module. This classification head comprises a Dropout layer with a probability of 0.5, followed by a 3D convolutional layer with a kernel size of 1 × 1, designed to categorize brain tumors into normal, benign, and malignant types. Finally, we applied a 3D average pooling layer to the model.

### A. HIGH-FREQUENCY INFORMATION RETENTION (HFIR)

**FIGURE 2.**
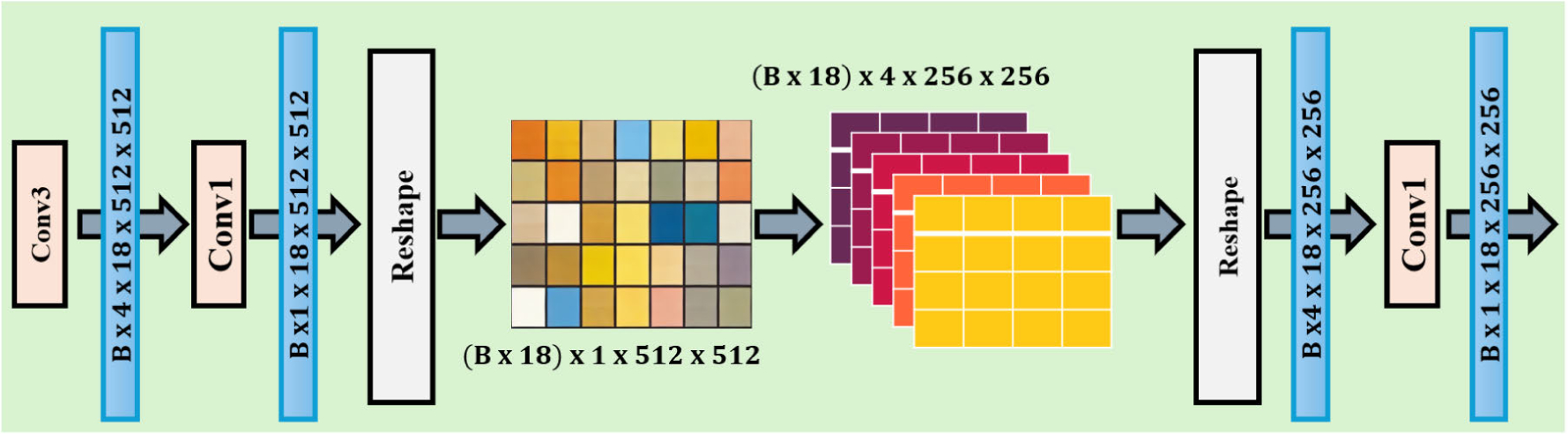
Detailed High-frequency information retention (HFIR) module. Here, *B*, Conv1, and Conv3 refer to the batch size, the convolution layer with a kernel size of 1 × 1, and the convolution layer with a kernel size of 3 × 3, respectively.

Accurate brain tumor classification relies on detailed information included in multimodal MRI scans of the brain. Therefore, preserving these details is crucial for this task, particularly for maintaining high-frequency information. Although maintaining high resolution throughout the network structure might seem intuitive, it significantly increases computational demand. However, data loss and performance degradation are inevitable outcomes of downsampling using convolution with stride or pooling algorithms. To tackle these issues, we used Pixel Unshuffle to reduce the image width (W) and height (H) to half their original sizes while simultaneously increasing the number of channels without losing any high-frequency information, as illustrated in Fig. 2.

In particular, in our trials, we used a 3 × 3 convolutional layer to extract shallow features $s\in R^{C\times D\times W\times H}$ for every T2-MRI image $x\in R^{1\times D\times W\times H}$, where D=18,W=512, and H=512. Subsequently, our architecture reduces the channel dimensions to $s\in R^{C/r^{2}\times D\times W\times H}$ through an additional 3 × 3 convolutional layer. Then, our architecture enlarges the feature map s using Pixel Unshuffle to $s\in R^{C\times D\times W/r\times H/r}$, where r=2, and then reshapes it to $s\in R^{\left(\left(C/r^{2}\right)*D\right)\times W\times H}$. Notably, we continued the practice of using pixel-wise displacement [38] by retaining high-frequency data.

### B. CNN-BASED BACKBONE

Various deep architecture models have been pre-trained on the ImageNet dataset, including notable examples such as ResNet [39], EfficientNet [40], DenseNet [41], Swin Transformer [42], Global Filter Network [43], FastViT [44], Res2Net [45], and Focal Modulation Network [46]. In our study, we employed a dual-branch architecture using 3D ResNet-18, which incorporates residual connections to facilitate effective training of deep models. ResNet-18 is particularly helpful in medical imaging applications, where the quality and quantity of data require sophisticated models to ensure high accuracy and reliability in diagnostic tasks. Our approach involves extracting feature maps from layer 4 (i.e., $f_{T2},f_{DWI}\in R^{512\times 4\times 8\times 8}$) of the 3D ResNet-18 for each branch. The model subsequently concatenated these feature maps and passed them through the ARA for further refinement.

### C. ADAPTIVE REGION ATTENTION (ARA)

**FIGURE 3.**
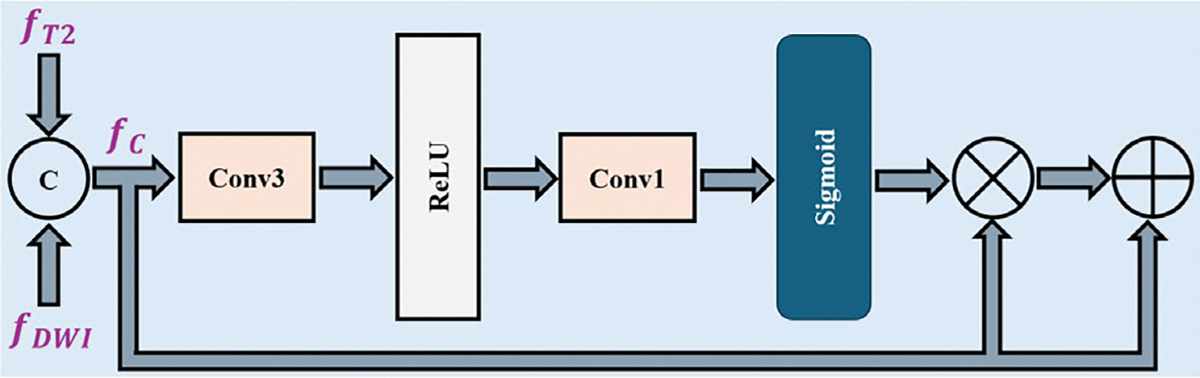
Schematic of the ARA module details. Here, $f_{DWI}$ and $f_{T2}$ refer to the CNN-derived features from layer 4 of the 3D ResNet-18 for MRI-DWI and MRI-T2, respectively, while $f_{C}$ represents the concatenation of $f_{DWI}$ and $f_{T2}$.

Intuitively, the high-level local feature $f_{T2}\in R^{P_{W}\times P_{H}\times C}$ and $f_{DWI}\in R^{P_{W}\times P_{H}\times C}$ originate from two distinct modalities and share the same spatial dimensions $P_{W}\times P_{H}$ and number of channels C. Consequently, they potentially lack correspondence and contain mismatched redundant and interference information. We introduced the ARA module, as shown in Fig. 3, to adeptly align and integrate the valuable insights from both modalities, designed to accentuate common, crucial features before further interaction and fusion.

In our design, the ARA module reshaped $f_{T2}$ and $f_{DWI}$ to produce $f_{T2}\in R^{P_{WH}\times C}$ and $f_{DWI}\in R^{P_{WH}\times C}$. Subsequently, the ARA module takes as input the concatenated features from both branches, denoted as $f:\left[f_{T2},f_{DWI}\right]\in R^{P_{WH}\times 2C}$, where [⋅,⋅] represents concatenation and $P_{WH}:P_{W}\times P_{H}$. The network processes the features through two fully connected (FC) layers: the first FC layer halves the feature dimension, and the second restores it to its original value. This technique achieves both goals by reducing the number of parameters and creating an information bottleneck effect. This effect compels the network to prioritize and pass only the most relevant information through the bottleneck, effectively sieving out irrelevant or redundant information. This design ensures that the network learns the most salient features of the data, thereby improving the model’s generalization capability. Subsequently, the model applied a sigmoid function to generate element-wise attention scores that highlighted the salient regions. The model incorporates a skip connection from the input to the output to enhance training stability. The proposed method mathematically represents the overall process as follows:

(1) $$\Gamma =\sigma \left(\Omega \left(f\Phi_{1}+b_{1}\right)\Phi_{2}+b_{2}\right)$$

(2) $$\left[\Gamma_{T2},\Gamma_{DWI}\right]=\Gamma$$

(3) $$\alpha_{T2}=\Gamma_{T2}⊗f_{T2}⊕f_{T2}$$

(4) $$\alpha_{DWI}=\Gamma_{DWI}⊗f_{DWI}⊕f_{DWI}$$

Here, $\Phi_{1}\in R^{2C\times (C/2)},\;\Phi_{2}\in R^{(C/2)\times 2C},\;b_{1}\in R^{1\times (C/2)}$, and $b_{2}\in R^{1\times 2C}$ define the weights and biases of the two fully connected layers; Ω denotes the ReLU activation function, and σ denotes the sigmoid function. The operator ⊗ indicates element-wise multiplication, and ⊕ indicates element-wise addition. The model divides $\Gamma \in R^{P_{WH}\times 2C}$ into $\Gamma_{T2}\in R^{P_{WH}\times C}$ and $\Gamma_{DWI}\in R^{P_{WH}\times C}$, and then uses them to obtain the attended region feature sequences $\alpha_{T2}\in R^{PWH\times C}$ and $\alpha_{DWI}\in R^{PWH\times C}$ from $f_{T2}$ and $f_{DWI}$, respectively.

## IV. EXPERIMENTAL RESULTS

### A. SETTING

We trained the model using the AdamW optimizer with a learning rate of 0.001, paired with a cosine annealing scheduler to facilitate learning rate decay. We used a consistent batch size of 8 throughout the training process. To optimize the model, we applied a cross-entropy loss function as the objective function. In addition, the model incorporated a Dropout layer with a probability of 0.5 before the final classification layer to mitigate overfitting. We initialized the 3D ResNet-18 backbone using random weights and trained the model from scratch. We adopted a leave-one-out cross-validation (LOOCV) strategy at the patient level for both training and evaluation. In each fold, we reserved all MRI scans (DW-MRI and T2-MRI) from a single patient for testing, while using data from the remaining patients for training. This approach ensured that no data from the same patient appeared in both the training and testing sets, thereby preventing data leakage and providing an unbiased assessment of the model generalizability across different individuals.

We implemented the system using the PyTorch framework in Python and conducted all experiments on a Windows-based machine equipped with 32 GB of RAM, a 20 GB NVIDIA graphics card, and a 12-core i7 processor.

### B. DATASET

In this study, our radiologists collected the dataset from 70 patients at the Diagnostic Radiology Department of Mansoura University Hospitals, Egypt. The Institutional Review Board (IRB) approved all procedures under protocol #R.21.09.1437.R1. Each patient underwent a standard MRI protocol on a 1.5T Philips Ingenia MRI scanner using a standard head coil. The dataset included four MRI sequences per subject: T1-weighted (T1-w), T2-weighted (T2-w), fluid-attenuated inversion recovery (FLAIR), and contrast-enhanced T1-weighted (T1CE) images. The acquisition parameters for the T1-w sequence included a repetition time (TR) of 580 ms, echo time (TE) of 15 ms, matrix size of 80 × 80, field of view (FOV) of 250 × 170 mm^2^, and slice thickness of 5 mm. The parameters for the T2-w images were TR: 4432 ms and TE: 100 ms. For the FLAIR sequence, TR: 10,000 ms, TE: 115 ms, and inversion time (TI): 2700 ms. The radiology team acquired contrast-enhanced T1-w images after intravenous administration of a gadolinium-based contrast agent using an automated injector at a dosage of 0.1 mmol/kg, flow rate of 2 mL/s, and maximum dose of 10 mL. Our radiologists divided the dataset into three diagnostic categories: (1) normal (no tumor detected), (2) benign (including meningioma and pituitary macroadenoma), and (3) malignant (including gliomas of various grades). The dataset consisted of 70 MRI samples divided into three classes: normal (20), benign (22), and malignant (28). Although the class distribution is not perfectly balanced, the imbalance reflects the natural availability of data in clinical settings. To mitigate potential bias, we report not only overall accuracy but also class-wise precision, recall, and F1-scores, which provide a fairer assessment of performance across all classes. We initially stored all MRI images in DICOM format and converted them to NIfTI format for subsequent preprocessing and model training. The dataset cannot be shared publicly due to privacy and institutional regulations; however, the IRB may grant access upon reasonable request.

### C. RESULTS

We evaluated the performance of the proposed system using various metrics, including accuracy (ACC), sensitivity (SEN), and specificity (SPE) for each brain tumor type. We conducted various experiments to evaluate the performance of the proposed method. Tables 2–4 summarize these experimental results. We began our experiments by comparing several well-known classification models with the proposed approach. As shown in Table 2, the proposed approach achieved superior performance on all evaluation metrics. We conducted a statistical analysis of the compared systems and Table 3 reports the results. To compute the 95% confidence intervals (CIs) for the mean F1-score and Cohen’s kappa, we applied bootstrap resampling to the predictions obtained from the LOOCV folds. Specifically, we generated 10,000 resampled test sets (with replacement) from the LOOCV predictions for all patients. These 10,000 samples do not represent unique patients but are resampled subsets that we used solely for statistical estimation.

Following the methodology proposed by Rajpurkar [47], we identified the 95% CIs as the range between the 2.5^th^ and 97.5^th^ percentiles of the bootstrap distributions. Furthermore, we computed the mean F1-score difference between our proposed model and the baseline methods using the same bootstrap sample. As shown in the “Diff” column of the Table 3, the 95% CIs of these differences exclude zero, providing statistical evidence that the proposed approach outperforms the competing models.

**FIGURE 4.**
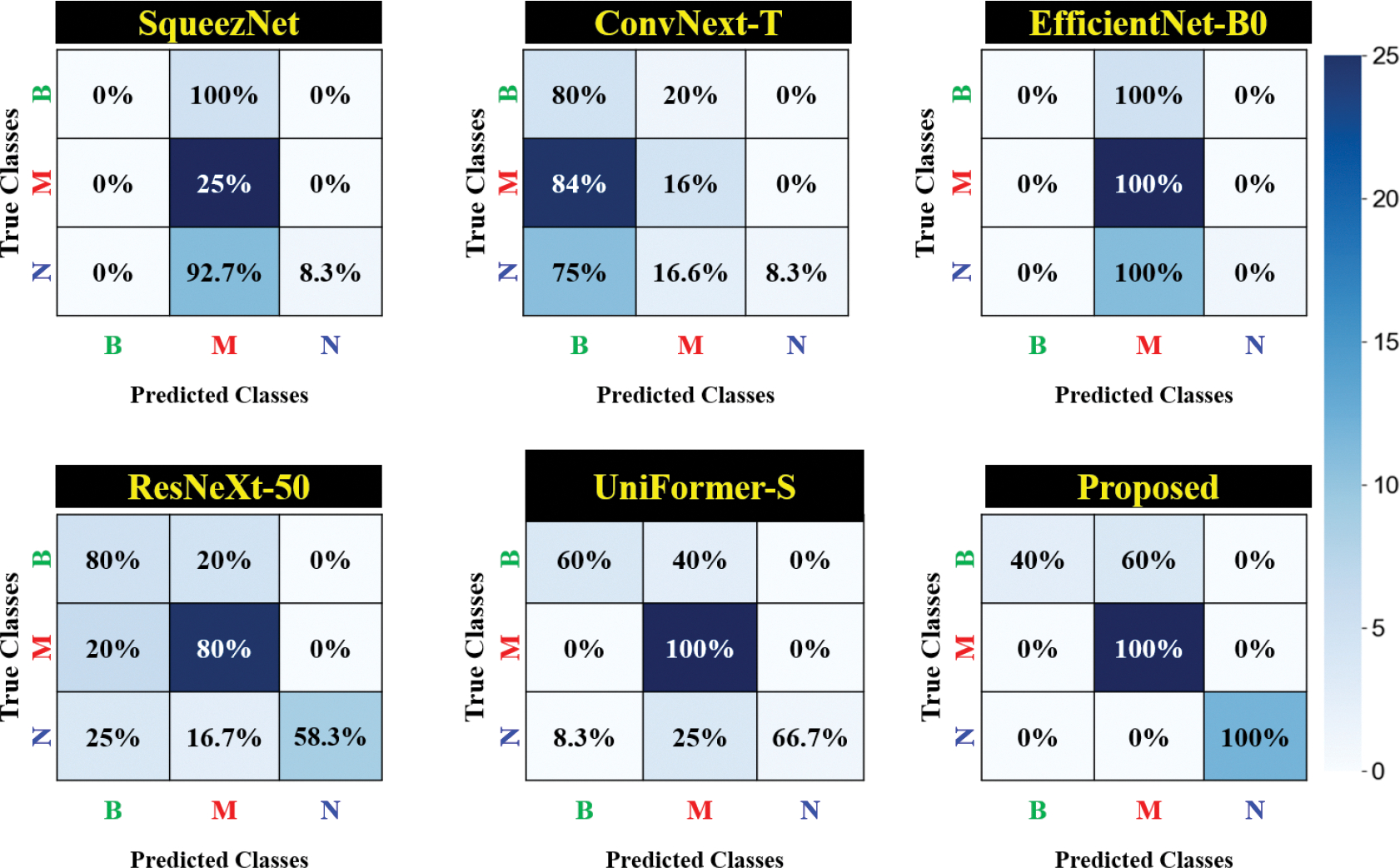
The confusion matrix for the proposed approach alongside other well-known multiclass classification models. The proposed method outperformed the other models in each class. Here “B”, “M”, and “N” stand for Benign, Malignant, and Normal, respectively.

**FIGURE 5.**
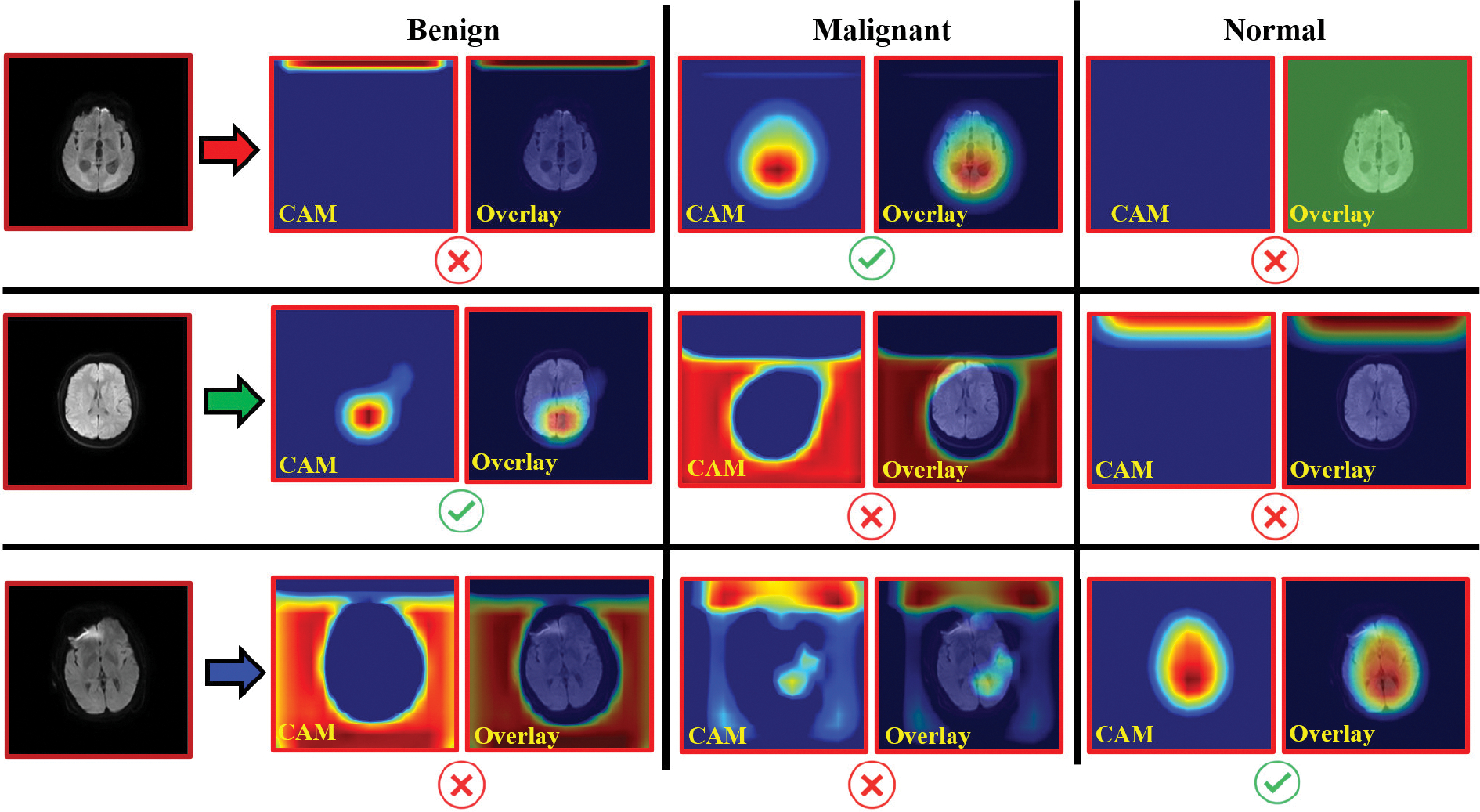
This figure illustrates the focus areas for the proposed model in classifying patients into different brain tumor types using grad-CAM. It presents a cross-section of DW-MRI with grad-CAM for each tumor class, accompanied by the corresponding overlaid Grad-CAM. The model predominantly emphasizes regions associated with malignant brain tumors, with intensity levels gradually decreasing as the distance from these regions increases.

An additional quantitative evaluation utilizes confusion matrices to provide a detailed breakdown of performance. Fig. 4 presents a confusion matrix comparing our approach with other models. The results illustrate that the proposed method outperforms all other models for each brain tumor type. For completeness, we illustrate the distinct regions within the input images emphasized by the proposed approach when classifying brain tumor types using Grad-CAM [52]. Fig. 5 presents the Grad-CAM overlay on cross-sectional DW-MRI images, highlighting the most influential area in the classification process.

Although the current dataset is limited to three-class classification (normal, benign, malignant), the multimodal integration and attention mechanisms in the proposed framework indicate the potential for subtype classification. The HFIR module’s retention of high-frequency details and the ARA module’s focus on shared tumor regions across modalities enable the model to capture subtle textural and diffusion variations. With an expanded dataset that includes subtype annotations (e.g., meningioma, pituitary macroadenoma, or glioma grades), we anticipate that the existing architecture can achieve high accuracy in subtype tasks, pending validation in future studies.

### D. ABLATION STUDY

While prior studies—such as those by Guo et al., Zhou et al., Xu et al., and Tabatabaei et al.—have reported strong performance using multimodal CNNs, attention mechanisms, or graph-based fusion models, their experimental designs often differ significantly. These differences include varying MRI modalities, reliance on 2D inputs, absence of 3D volumetric representation, and dependence on pre-trained feature extractors and shallow architectures. Furthermore, several approaches focus solely on segmentation or binary classification tasks using limited datasets. In contrast, our study introduces a novel full-volume dual-branch 3D CNN architecture enhanced by HFIR and ARA modules and designed specifically for multimodal brain MRI fusion. To fairly assess the contribution of each architectural component, we performed an internal ablation study under consistent training settings, rather than benchmarking against heterogeneous external baselines.

To assess the contribution of each component of the proposed approach, we conducted an ablation study, with the results presented in Table 4. In the first two scenarios, we evaluated the MRI data without integrating HFIR or ARA individually. First, we employed concatenated DW-MRI data (i.e., b0, b500, and b1000) as the inputs for the 3D ResNet-18 encoder. The system achieved an accuracy of 85.71% (first row of Table 4). Subsequently, the use of T2-MRI data alone achieved an accuracy of 83.33% (second row of Table 4). After applying the HFIR to downsample the T2-MRI images from 18×512×512 to 18×256×256, the 3D ResNet encoder achieved a similar accuracy. Next, we tested a multi-branch multimodality model, where the first branch extracted features from the HFIR-based downsampled T2-MRI images, and the second branch extracted features from the DW-MRI data. The proposed module concatenated the features from both branches and input into the classification head, achieving an accuracy of 78.57% (fourth row of Table 4). In the fifth row of Table 4, the model input features from both branches into the ARA module to align and highlight common features between them, resulting in an accuracy of 92.86%.

## V. DISCUSSION

This ongoing field of study explores the development of AI-based on streamlined computational approaches for brain tumor diagnosis and detection. This advancement in AI-powered diagnostics holds great promise for improving patient outcomes by allowing earlier detection and more effective treatment plans [53]. Recent developments in AI/ML methods provide a viable path for creative detection solutions that employ brain MRI images in response to this demand. This study aims to provide a robust multimodal analytical tool backed by a degree of explainability and interpretability for brain tumor identification. We propose a comprehensive and innovative learning architecture that integrates multiple learning modules to analyze brain MRI images for a more accurate brain tumor diagnosis.

The proposed pipeline is a multimodal learning architecture that integrates a dual-branch 3D CNN encoder, HFIR, and ARA modules. We introduced the HFIR module to preserve the intricate details essential for accurate brain tumor classification by maintaining high-frequency information. Furthermore, the employed ARA-based strategy not only leverages the various perspectives provided by each modality but also significantly enhances the model’s performance by accentuating crucial modality features prior to further interaction and fusion. Since these models together, Table 2 indicates an overall accuracy of 92.86%. Our model performed better than DL architectures [49], [50] and modern transformer-based architectures [51], underscoring its potential as a valuable tool for brain tumor diagnosis. Additionally, bootstrap analysis of the results obtained by the proposed and competing methods documents the robustness of the proposed architecture. As shown in Table 3, the 95% confidence intervals for both the F1-score and Cohen’s kappa indicate that our method is statistically significantly superior to the other models. These results demonstrate the efficacy of the proposed system in achieving improved performance and underscore its robustness and capabilities.

Furthermore, we conducted an ablation analysis, broke down the pipeline under various evaluation scenarios, and summarized the results in Table 4 using ACC, SEN, and SPEC metrics. Typically, we evaluated the contribution of each modality to the overall accuracy. As indicated in Table 4, the DWI-based diagnosis yielded a very low sensitivity of 63.89%. This low sensitivity is likely due to concatenated DWI data with varying b-values, which may have introduced noise due to the differences in these b-values. The T2-MRI-based diagnosis achieved a sensitivity of 65.00%. The use of three b-value volumes in DWI compared with a single T2 volume contributed to its higher accuracy compared to T2-MRI. Our multi-branch multimodality model, developed with the HFIR module, improved the performance (i.e., sensitivity) to 70.33%. The features from both branches with ARA enhancement increased the system sensitivity to 80.00%. This ablation study highlights the importance of ARA before feature fusion to align commonalities between various MRI-derived features.

The superior performance of the proposed multimodal architecture stems from the effective integration of the HFIR and ARA modules. Unlike conventional models such as SqueezeNet, ResNet-50, EfficientNet-B0, and Uniformer-S, which often apply generic processing to entire the brain volume, our approach leverages HFIR to preserve diagnostic details during T2-MRI downsampling. ARA focuses on clinically relevant regions that are shared across modalities within the proposed model. This targeted feature enhancement improves robustness against class imbalance and dataset limitations, as shown in Fig. 5 by Grad-CAM visualizations. In addition, as shown in Table 4, individual modality performance is strong (83.71% for DW-MRI and 83.33% for T2-MRI), but their integration within the proposed dual-branch framework significantly boosts the accuracy to 92.86%. The ablation study further confirmed the substantial contributions of HFIR and ARA to this improvement, demonstrating their critical role in outperforming existing methods.

Generally, there is a trade-off between system complexity (in terms of the number of parameters) and increased accuracy. Our pipeline illustrates that the shallow design of ResNet-18, paired with enhanced feature fusion, enables more effective learning and higher accuracy. Furthermore, openness and interpretability are essential for building confidence and enabling more informed diagnoses. Therefore, our pipeline incorporates the Grad-CAM method to emphasize the MRI areas that greatly affect pipeline predictions. Fig. 5 illustrates these results in detail. The first column displays sample images from the many classes (benign, normal, malignant), and the next column shows the Grad-CAM heatmap projected for the three classes superimposed on grayscale images. The model marks the regions that most strongly affect its predictions with heightened intensity. This all-encompassing visualization method improves the interpretability and transparency of the multimodal model’s decision-making process, in addition to revealing the particular elements guiding the model predictions.

The current study presents a viable method for classifying brain tumors. Nonetheless, certain limitations of this study offer opportunities for future research. First, the model considers MRI image data, which may cause it to ignore essential information in pathological or clinical feature data. Second, we utilized a private dataset in this study that was small and affected by class imbalance. Third, the system processes the whole brain volume, and there is no mechanism in the model to specifically identify and concentrate on important brain ROIs in the images. Ultimately, this study offers a comprehensive diagnostic tool that distinguishes between normal, abnormal, and benign cases. However, future studies should focus on diagnosing different brain tumor subtypes to expand the scope of this study and enhance its applicability and usefulness. Future studies that overcome these constraints may provide useful information for clinical decision-making and enhance the algorithm’s classification performance.

## VI. CONCLUSION AND FUTURE WORK

In this study, we introduced a novel multimodal adaptive inter-region attention-guided framework for brain tumor classification, integrating DW-MRI and T2-MRI through the HFIR and ARA modules. The HFIR module preserved high-frequency details while downscaling resolution to reduce information loss, and the ARA module emphasized shared tumor-relevant regions across modalities within a dual-branch 3D ResNet-18 architecture. On a locally curated dataset of 70 patients categorized into normal, benign, and malignant classes, the proposed framework achieved an accuracy of 92.86%, sensitivity of 80.00%, and specificity of 94.12%. Statistical analyses—using bootstrap-derived 95% confidence intervals for F1-scores—confirmed significant outperformance over other methods. Meanwhile, Grad-CAM visualizations highlighted diagnostically salient regions, thereby enhancing interpretability for clinical adoption.

While these results validate the framework’s efficacy, validation on larger and more heterogeneous datasets is essential to assess robustness against domain shifts such as scanner strength (e.g., 1.5T vs. 3T) and acquisition protocols. Future work will therefore focus on comprehensive evaluations using diverse multimodal datasets, potentially incorporating T1, T1c, and FLAIR sequences to extend the framework toward subtype classification and glioma grading. We also aim to expand the system to more complex clinical tasks, including tumor detection and segmentation, leveraging the HFIR module for modality-agnostic preprocessing and the ARA module for cross-modality feature alignment. We will rigorously assess the framework through multi-fold cross-validation and direct comparisons against state-of-the-art architectures across diverse modalities.

## Figures and Tables

**TABLE 1.** Summary of recent literature work for brain tumor detection.

Ref	Method	Dataset	Performance	Limitation	Focus Area
[26]	Cross-modality guided ResNet backbone with dual attention for brain tumor grading	BraTS 2018 and 2019	Accuracy of 97.90% for BraTS 2018 and 97.00% for BraTS for 2019	Performance varies across modalities; lacks high-frequency detail preservation	Multimodal MRI input
[27]	MMIDFNet for improving glioma subtype classification accuracy	CPMRP	87.80% accuracy	Limited tumor type scope, lacks multi-scale fusion and volumetric modeling.	Multimodal MRI Input
[28]	Dual Path Network based on MMD-Net for brain tumor segmentation	BraTS 2015	92.00% precision	Inadequate spatial context and receptive field, lacks adaptive regional attention	Multimodal MRI Input
[29]	Introducing Patch Individual Filter for CNNs	UK Biobank, ADNI, Private MS	89.06% for UK Biobank, 84.43% for ADNI, and 80.92%	Not optimized for tumor-specific learning; lacks frequency-aware feature retention	MRI Frequency and Processing
[31]	ResNet50 with KMeans and SGLDM, including Grad-CAM for classification	Br35H	Training accuracy of 99.25% and validation accuracy of 99.50%	Heavily data-dependent, no volumetric or multimodal analysis.	MRI Frequency and Processing
[37]	UNet and ResNet brain segmentation framework	Figshare	95% accuracy	Uses 2D slices; lacks full 3D feature learning and multimodal integration	MRI Frequency and Processing
[30]	Five block dense autoencoder (BT-AutoNet) with 7 Noise Filters, RDUNet, and BBAutoNet for denoising	Local (Nanfang Hospital), Three Noise datasets	Average SSIM of 94.58% for all noises and datasets	Denoising is limited to fixed noise types; lacks generalizability to tumor classification.	MRI Frequency and Processing
[32]	Local Pixel Inhomogeneity Factor (LPIF) for CNN	BRATS 2013	Accuracy 99.46% for DS1 and 98.63% for DS2	High computational cost, lacks modality-aware attention and hierarchical fusion	MRI Frequency and Processing
[17]	Hybrid CNN-Transformer with bidirectional fusion	Figshare	98.59–99.30% accuracy	No spatial alignment across modalities; lacks high-frequency and deep-level integration	Attention Mechanisms or Feature Refinement
[21]	Multi-level Attention Network (MANet)	BRATS 2018, FigShare	96.51% accuracy	Focused only on malignant tumors; no adaptive feature enhancement	Attention Mechanisms or Feature Refinement
[33]	MobileNetvl+v2 with attention + LSTM fusion	Br35H	96.88% accuracy	CNN backbone lacks depth; limited by dataset size and diversity	Attention Mechanisms or Feature Refinement
[34]	Custom 3D UNet CNN for segmentation	BRATS 2018 and 2019	Average DSC of 85.18% for BraTS 2018 and 83.68% for BraTS 2019	No ARA, lacks explicit preservation of frequency features.	3D Dual-Branch CNN Architecture
[36]	2D Parallel, Attention-Enhanced customized CNN: ResNet50 with VGG16	Figshare	98.04% accuracy	Relies on pre-segmented tumors for accuracy, lacks full 3D feature fusion and HFIR.	3D Dual-Branch CNN Architecture
[35]	Custom 3D CNN with multi-branch attention mechanism	BraTS 2020 and 2021	Average DSC of 87.20% for BraTS 2020 and 87.03% for BRATS 2021	Employs only early fusion; no frequency or region-specific module	3D Dual-Branch CNN Architecture

***Abbreviations:*** MANet: Multi-level Attention Network; CNN: Convolutional Neural Network; ADNI: Alzheimer’s Disease Neuroimaging Initiative; LPIF: Local Pixel Inhomogeneity Factor; MS: Multiple Sclerosis; LSTM: Long Short-Term Memory; BraTS: Brain Tumor Segmentation Challenge; MMIDFNet: Multimodal MRI Image Decision Fusion Network; CPMRP: Computational Precision Medicine: Radiology-Pathology; MMDNet: Multimodal DenseNet; SGLDM: Spatial Gray Level Dependence Matrix; DSC: Dice Similarity Coefficient; Grad-CAM: Gradient-weighted Class Activation Mapping; SSIM: Structural Similarity Index Measure; RDUNet: Residual Dense Network; BBAutoNet: Block-based Autoencoder Network; VGG: Visual Geometry Group.

**TABLE 2.** Comparison of the proposed approach with several well-known multiclass classification models using accuracy (ACC), sensitivity (SEN), and specificity (SPE). Here, “T” indicates the tiny version of the respective model, and “S” denotes the small one.

Model	ACC (%)	SEN (%)	SPE (%)
SqueezeNet [48]	61.90	36.11	68.63
ConvNext-T [49]	21.43	34.78	67.09
EfficientNet-BO [40]	59.52	33.33	66.67
ResNeXt-50 [50]	73.81	72.78	86.91
UniFormer-S [51]	85.71	75.56	89.30
**Proposed**	**92.86**	**80.00**	**94.12**

**TABLE 3.** Comparison of the proposed approach with several well-known models based on F1-score, Cohen’s kappa, and their differences.

Model	F1-Score (%) (95% CI)	Cohen Kappa (%) (95% CI)	Diff
SqueezeNet [48]	30.73 (20.00, 53.33)	7.13 (0.00, 34.78)	51.26 (50.80, 51.71)
ConvNext-T [49]	18.82 (3.51, 38.7)	1.33 (0, 17.42)	63.16 (62.7, 63.60)
EfficientNet-B0 [40]	25.3 (18.39, 41.67)	0.00 (0.00,0.00)	56.35 (55.95, 56.73)
ResNeXt-50 [50]	65.82 (43.21, 88.57)	54.75 (24.32, 83.59)	16.16 (15.67, 16.65)
UniFormer-S [51]	76.37 (48.21, 100.00)	70.57 (38.24, 100.00)	5.01 (5.09, 6.14)
**Proposed**	**81.98 (62.21, 100.00)**	**85.69 (62.21, 100.00)**	–

**TABLE 4.** An ablation study of key components of the proposed 3D model.

DW-MRI	T2-MRI	3D ResNet	HFIR	ARA	ACC (%)	SEN (%)	SPE (%)
✓		✓			85.71	63.89	89.08
	✓	✓			83.33	65.00	87.12
	✓	✓	✓		83.33	70.33	87.12
✓	✓	✓	✓		78.57	55.56	82.35
✓	✓	✓	✓	✓	**92.86**	**80.00**	**94.12**
